# Fluorescence Polarization and Fluctuation Analysis Monitors Subunit Proximity, Stoichiometry, and Protein Complex Hydrodynamics

**DOI:** 10.1371/journal.pone.0038209

**Published:** 2012-05-30

**Authors:** Tuan A. Nguyen, Pabak Sarkar, Jithesh V. Veetil, Srinagesh V. Koushik, Steven S. Vogel

**Affiliations:** Section on Cellular Biophotonics, Laboratory of Molecular Physiology, National Institute on Alcohol Abuse and Alcoholism, National Institutes of Health, Rockville, Maryland, United States of America; Mount Sinai School of Medicine, United States of America

## Abstract

Förster resonance energy transfer (FRET) microscopy is frequently used to study protein interactions and conformational changes in living cells. The utility of FRET is limited by false positive and negative signals. To overcome these limitations we have developed Fluorescence Polarization and Fluctuation Analysis (FPFA), a hybrid single-molecule based method combining time-resolved fluorescence anisotropy (homo-FRET) and fluorescence correlation spectroscopy. Using FPFA, homo-FRET (a 1–10 nm proximity gauge), brightness (a measure of the number of fluorescent subunits in a complex), and correlation time (an attribute sensitive to the mass and shape of a protein complex) can be simultaneously measured. These measurements together rigorously constrain the interpretation of FRET signals. Venus based control-constructs were used to validate FPFA. The utility of FPFA was demonstrated by measuring in living cells the number of subunits in the α-isoform of Venus-tagged calcium-calmodulin dependent protein kinase-II (CaMKIIα) holoenzyme. Brightness analysis revealed that the holoenzyme has, on average, 11.9±1.2 subunit, but values ranged from 10–14 in individual cells. Homo-FRET analysis simultaneously detected that catalytic domains were arranged as dimers in the dodecameric holoenzyme, and this paired organization was confirmed by quantitative hetero-FRET analysis. In freshly prepared cell homogenates FPFA detected only 10.2±1.3 subunits in the holoenzyme with values ranging from 9–12. Despite the reduction in subunit number, catalytic domains were still arranged as pairs in homogenates. Thus, FPFA suggests that while the absolute number of subunits in an auto-inhibited holoenzyme might vary from cell to cell, the organization of catalytic domains into pairs is preserved.

## Introduction

**Figure 1:**
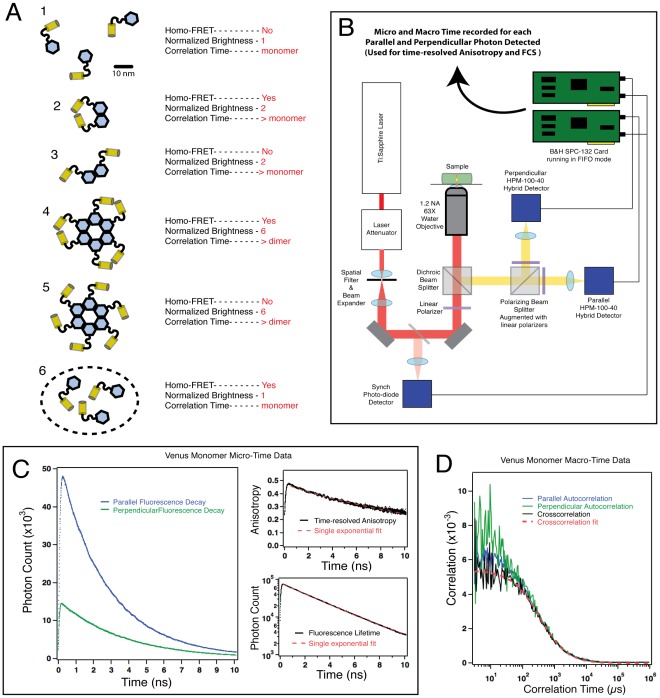
Fluorescence Polarization and Fluctuation Analysis: A method for studying the structure of protein complexes. (A) Six possible structures for a hypothetical protein (Blue hexagon) tagged with Venus (yellow cylinder). Example 1 depicts monomers. Examples 2 and 3 depict dimers. Attached Venus molecules are in close apposition for example 2 but not for example 3. Examples 4 and 5 depict hexamers. Pairs of attached Venus molecules are in close apposition for example 4 but not for example 5. Example 6 depicts monomers that are confined to a sub-compartment (dashed line) where they are expressed at a high concentration. (B) Schematic for microscope to measure FPFA. (C) Micro-time data measured for a homogenate prepared from cells expressing Venus monomers are used to calculate fluorescence lifetime histograms for photons detected by either the parallel or perpendicular hybrid-detectors as a function of time after the laser excitation pulse (left panel). These two decay curves are then used to calculate the time-resolved anisotropy (right panel top), or fluorescence lifetime (right panel bottom) of Venus monomers. Red dashed lines show fitting to a single exponential model. (D) Macro-time data measured for a homogenate prepared from cells expressing Venus monomers are used to calculate auto- and cross-correlation functions for photons detected in the parallel and perpendicular hybrid-detectors. A single diffusible-component 3-D Gaussian model was used to fit the cross-correlation (red dashed line), and to estimate the correlation time and the average number of molecules in the observation volume. The excitation power used was 10.2 mW.

In cells, proteins rarely function individually; typically they interact with other proteins to mediate cellular processes. Determining the structure and dynamics of protein complexes in cells requires noninvasive high-resolution spatial and temporal methods, such as Förster resonance energy transfer (FRET) microscopy [1], [2], [3], [4], [5], [6]. Because the efficiency of FRET is inversely proportional to the 6^th^ power of the distance separating fluorescent donors and acceptors [2], [3], [7], FRET has been used to study protein interactions in cells. Energy transfer between proteins tagged with either the same fluorophore (homo-FRET [8], [9], [10], [11], [12]) or spectrally distinct fluorophores (hetero-FRET [2], [3]) can be monitored using time correlated single photon counting (TCSPC [13]) to measure dynamic changes in fluorescence polarization or intensity respectively. However, the absence of FRET does not necessarily mean that proteins are not in a complex because the distance separating fluorophores in a complex may be larger than 10 nm (or their dipole orientation may be unfavorable). Similarly, a positive FRET signal may arise from non-specific FRET caused by protein over-expression rather than complex formation. These limitations are illustrated in figure 1A, in which six possible arrangements of a fluorescent protein-tagged subunit are depicted. In this diagram homo-FRET is expected for protein complexes if their attached fluorophores (yellow cylinders) are in close proximity. Thus, homo-FRET would not be observed for examples 1, 3, and 5 (even though example 3 is a dimer and example 5 is a hexamer), but FRET should be observed in examples 2 and 4. Furthermore, FRET measurements alone cannot differentiate between example 2 (a dimer) and example 4 (a hexamer). Homo-FRET should also be observed for example 6, monomers in close proximity. One potential way to differentiate all six subunit arrangements depicted in figure 1A is to measure homo-FRET and Fluorescence Correlation Spectroscopy (FCS) simultaneously.

Unlike FRET, FCS monitors the motion of fluorophore-tagged proteins, not their proximity [14], [15], [16], [17]. In a FCS experiment temporal fluctuations of fluorescence intensity emanating from a small volume (typically less than 1 fl) are measured as individual protein complexes traverse the observation volume [16], [17]. Auto- and cross-correlation analysis [16], [17], [18] of theses fluctuations can then reveal the average number of fluorescent molecules in the observation volume, <N>. Molecular brightness, the average number of photon counts per second per fluctuating molecule (cpsm), is obtained by taking the ratio *<k>*/<N>, where *<k>* is the average fluorescent count rate [17]. With control experiments to measure the brightness of free fluorophore, the molecular brightness, can be used to calculate the *Normalized Brightness* - the average number of fluorophores in a protein complex. If each subunit is tagged with only a single fluorophore, the normalized brightness will reflect the number of subunits in a complex. This is called *Brightness Analysis* [19]. In addition, FCS can also determine the average amount of time a protein complex remains in the observation volume [17]; a value related to the lateral diffusion coefficient of the complex, itself a function of viscosity, mass and hydrodynamic volume (and hence the conformation of the complex). Another technical advantage of FCS analysis is that single molecule fluctuations in the fluorescent intensity can only be observed at very low fluorophore concentrations (typically nano-molar) [17], thus, many of the problems associated with over expression of exogenous proteins cannot exist in successful FCS experiments. By considering *both* the presence (or absence) of homo-FRET, *as well as* the normalized brightness, all 6 examples depicted in figure 1A could be differentiated. Furthermore, often these interpretations could be corroborated using the lateral diffusion time, an additional parameter measured.

While FRET and FCS are usually considered alternative approaches for studying protein complexes [20], figure 1A illustrates that combining them could be advantageous. New instrumentation for TCSPC can now record both *micro-time* (the elapsed time between photon detection and the laser excitation pulse) and *macro-time* (the elapsed time between the start of an experiment and when an individual photon is detected) [13], [21]. This technology enables combining FCS (based on macro-time data) and either hetero- or homo-FRET (which both use micro-time data) with high photon efficiency. While combining hetero-FRET and FCS is problematic (donor emission may bleed into the acceptor signal, and hetero-FRET itself will cause reciprocal intensity fluctuations in donor and acceptor channels) [20], combining homo-FRET and FCS is more straightforward (because only a single fluorophore is used). An additional benefit of combining homo-FRET and FCS is that spectral bandwidth for fluorophore excitation and emission is kept narrow enabling the use of other fluorophores in more complicated multiplexed experiments.

Here we describe a new method, Fluorescence Polarization and Fluctuation Analysis (FPFA), that combines Förster resonance energy transfer microscopy (FRET) [1], [2], [3], [4], [5], [6] and Fluorescence Correlation Spectroscopy (FCS) [14], [15], [16], [17] as a tool to study the structure of protein complexes inside living cells. We developed this method specifically to characterize the structure of the calcium-calmodulin dependent protein kinase-II (CaMKII) holoenzyme under physiological conditions. CaMKII is an excellent example of a protein whose function depends on protein-protein interactions. CaMKII subunits assemble to form a multimeric kinase involved in memory and synaptic modulation [22], [23]. The number of subunits that comprise the holoenzyme is uncertain with estimates ranging from 3 to 14 [24], [25], [26], [27], [28], [29], [30]. In cells the kinase is transiently activated in response to a rise in intracellular calcium. Calcium binds to calmodulin (CaM) to form calcium/calmodulin (Ca^2+^/CaM) [23], and Ca^2+^/CaM in turn binds to the regulatory domain of CaMKII. Ca^2+^/CaM binding to CaMKII is thought to trigger a large conformational change that opens the substrate-binding site on the catalytic domain [30]. Unlike most other biological calcium sensors, CaMKII is sensitive to the frequency of calcium spikes [31]. This unique ability is thought to be an emergent feature of its holoenzyme organization [32], and is thought to play a role in memory storage [22]. Little is known about the structure or stoichiometry of the holoenzyme under physiological conditions, in part because few methods exist to monitor the structure of protein complexes in cells.

CaMKII monomers have three domains, the N-terminal catalytic domain, a regulatory domain, and a C-terminal association domain responsible for holoenzyme oligomerization [23], [33]. In the auto-inhibited holoenzyme, catalytic domains are thought to dimerize [10], [30], [34], resulting in a holoenzyme structure with multiple catalytic domain pairs distributed around a central association domain core. X-ray crystallography was first to indicate that isolated CaMKIIα catalytic domains are arranged as dimers [34], but the validity of this conclusion is controversial, as this dimeric structure was not observed in X-ray data sets for isolated catalytic domains from several other CaMKII isoforms [30]. Nonetheless, analytic ultracentrifugation of isolated catalytic domains from multiple isoforms did detect catalytic domain dimerization [30], as did homo-FRET experiments with fluorescent protein-tagged CaMKIIα in hippocampal neurons [10]. Catalytic domain pairing was not observed in a recent crystal structure of a mutated CaMKIIβ7 holoenzyme. In this new model for the holoenzyme structure individual catalytic domains are docked onto the central association domain core as monomers to form a compact holoenzyme structure [35]. Because Ca^2+^/CaM binding sites on the regulatory domain are inaccessible in this compact structure, it was proposed that a more extended auto-inhibited structure must also exist to allow for activation. Mutation in the CaMKII regulatory domain (TT305/306DD for the α isoform) are known to prevent Ca^2+^/CaM from binding to CaMKII [36], and based on this new model are now predicted to also disrupt the docking of catalytic domains to the association domain core [35]. Thus it is possible that catalytic domains might form pairs after they un-dock from the association domain core. It should be noted that unlike most CaMKII isoforms, the CaMKIIβ7 isoform had dramatically reduced levels of catalytic activity under physiological conditions [35], [37]. Thus, it is unclear if this new model for the intact CaMKIIβ7 holoenzyme reflects the structure of active CaMKII isoforms in cells.

In this study we develop a new method for characterizing protein complexes in living cells, FPFA. We first validate this method with two sets of Venus [38] based control constructs, and then apply FPFA to simultaneously measure the number of subunits in the CaMKIIα holoenzyme in cells, as well as the organization of its catalytic domains.

## Results

The experimental setup used for linear-polarized two-photon excitation of fluorophores in a small focal volume is shown in figure 1B. Both micro- and macro-times of emitted photons detected through orthogonally oriented polarization pathways (parallel and perpendicular) were recorded. Calibration experiments with fluorescein and Venus monomers were used to identify excitation powers *in vitro* where bleaching of Venus was not detected, and to then estimate the volume and dimensions of our observation volume under these conditions (see materials and methods). For *in vitro* measurements laser powers ranging from 9.0–10.2 mW were used. Time-resolved anisotropy (TRA) and lifetime were calculated from histograms of parallel and perpendicular detector micro-times (Fig 1C), while polarization corrected correlation times and brightness were calculated by fitting the cross-correlation of these detectors macro-time signals [39] (Fig 1D).

### Validation of FPFA

**Figure 2:**
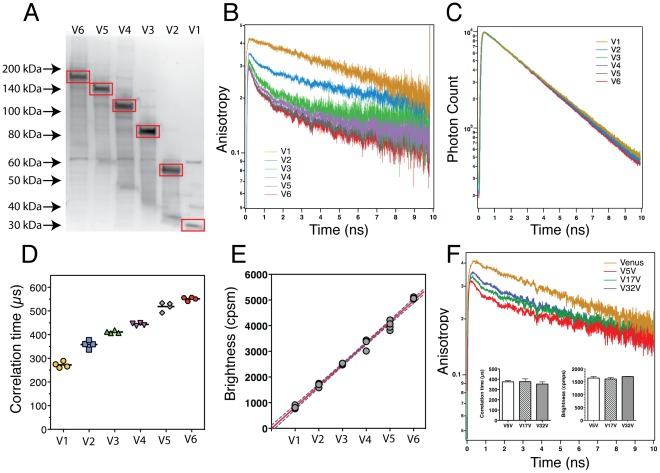
FPFA Validation. (A) Western blot of homogenates prepared from cells expressing Venus monomers (V1), dimers (V2), trimers (V3), tetramers (V4), pentamers (V5) and hexamers (V6) using a GFP specific antibody. Red boxes outline the immuno-reactive components corresponding to the expected molecular weights of these concatemers. (B) Time-resolved anisotropy (TRA) recorded from homogenates prepared from cells expressing V1–V6. The data are plotted on a semi-log scale to highlight the single exponential decay of V1 as compared to the multi-exponential decays of V2–V6. (C) Fluorescence lifetime decays of V1–V6. (D) The correlation times for V1–V6 are plotted. Bars represent mean values for each construct (n = 4). The excitation power used was 9.0 mW. (E) The brightness values for V1–V6 are plotted as a function of the number of Venus molecules in each construct. Red line indicates fit to a linear model with dashed blue lines indicating the 95% confidence bands. (F) TRA of three Venus dimers, V5V, V17V, and V32V, where 5, 17, and 32 indicates the number of amino-acids in the linker separating the two fluorophores. The TRA of Venus monomers are also shown to highlight that all three of these constructs had a fast decay component due to homo-FRET. Insets show the correlation times and brightness of these samples (mean±SD, n = 3).

FPFA was validated using 6 fluorescent oligomers composed of between 1 and 6 concatenated Venus molecules (V1–V6). Venus, a yellow GFP spectral variant, was used because it matures rapidly [38], and has a Förster distance of 5.3 nm [40] supporting efficient homo-FRET. To avoid nonspecific aggregation, a monomeric variant of Venus was used in all experiments [41]. Immunoblot analysis using a GFP specific antibody confirmed that the molecular weight of these constructs increased linearly with the number of Venus molecules (Fig 2A). Micro-time data was used to calculate the TRA (Fig 2B) of homogenates prepared from cells expressing V1–V6. The V1 anisotropy decayed as a single-exponential (τ = 15.3±0.8 ns). Like V1, V2–V6 also had a slow decay component (τ>15 ns), as well as additional fast decay components occurring primarily within the first 2 ns. The similarity between the V1 decay constant and the rotational time constant of purified Venus (16.4±0.6 ns) [42] indicates that V1 is a monomer in solution. The additional *fast* depolarization observed for V2–V6, much faster than monomer rotation, is the hallmark of homo-FRET between fluorescent proteins [10], [11]. While the amplitude of these fast anisotropy decay components is a function of the number of Venus molecules exchanging energy by FRET [10], [11], the similarity between the V4–V6 anisotropy decay curves reveals a major limitation for using homo-FRET alone to estimate subunit stoichiometry in a complex with four or more subunits.

The same micro-time data was also used to calculate the lifetime of Venus in these control constructs (Fig 2C). The lifetime of Venus was 3.1±0.0 ns, similar to values measured previously (3.03±0.01 ns) [42], and its lifetime was not appreciably altered by concatenation or by homo-FRET. Because the photon count rate emitted from a molecule depends on the molecule’s lifetime, this observation also indicates that subsequent FCS based brightness analysis will not be significantly influenced by homo-FRET. Thus, the primary source of intensity fluctuations in the FPFA experiments described here should be the *diffusion* of Venus-tagged molecules in and out of the excitation volume.

Macro-time data was used to calculate the cross-correlation between photons detected in parallel and perpendicular channels [39]. Cross-correlations for Venus concatemers were all well fit using a single-component 3-dimensional Gaussian model [17], and these fits were used to derive the correlation time and the average number of diffusing molecules in the observation volume. The later was used to normalize the photon count rate to calculate molecular brightness as mentioned earlier. Figures 2D & E show the correlation time and molecular brightness obtained for each concatemer. A systematic increase in correlation time was observed as the number of Venus molecules per concatemer increased, but as expected, correlation time was not very sensitive to changes in mass. Molecular brightness also increased with the number of Venus molecules in a concatemer, but unlike the correlation time, molecular brightness was linearly proportional to the number of Venus molecules in a concatemer (Fig 2E). Thus, while changes in correlation time may serve as an indicator of complex formation, the ratio of the brightness of a construct and the brightness of a Venus monomer can directly reveal the number of Venus molecules in a construct. This normalized brightness analysis can potentially be used to specify the number of fluorophore-tagged subunits in a protein complex, as will be shown later when FPFA is used to investigate the holoenzyme structure of Venus-tagged CaMKII.

Because FPFA can measure homo-FRET, it should be able to detect structural changes that FCS cannot. To test this capability, FPFA was performed on three different Venus dimers, V5V (the V2 construct), V17V and V32V, where 5, 17 and 32 amino acids linkers separate Venus molecules respectively. TRA analysis revealed that these 3 dimers all had fast decay components indicative of homo-FRET [11] (Fig 2F). The decay rate of these fast components should be proportional to the FRET transfer rate, itself a function of the distance between Venus molecules [11]. Consistent with this, the anisotropy of V5V decayed fastest while V32V decayed the slowest. In contrast, the correlation times and brightness of V5V, V17V, and V32V were comparable (Fig 2F inset).

### FPFA Studies of CaMKIIα Holoenzyme in vitro

To measure the number of subunits in the CaMKIIα holoenzyme with FPFA, cells were transfected with DNA encoding Venus-CaMKIIα (V-CaMKIIα). Previous studies have shown that tagging the N-terminus of CaMKII with GFP does not alter its catalytic activity, auto-phosphorylation, or the ability of the kinase to assemble [36], [43]. Furthermore, we have shown that V-CaMKIIα expressed in neurons undergoes a change in anisotropy correlated with calcium influx through NMDA receptors [10]. The next day, transfected cells were harvested and homogenates prepared for FPFA. Supernatants from homogenates of cells expressing V-CaMKIIα had a normalized brightness of 10.2±1.3 (n = 5, mean±SD) (Fig 2A). FPFA experiments were repeated using V-CaMKIIα[TT305/306DD] a mutant that cannot bind Ca^2+^/CaM to determine if the auto-inhibited holoenzyme is also a decamer. V-CaMKIIα[TT305/306DD] had a mean normalized brightness of 10.7±1.3 (n = 5, mean±SD). These values were not statistically different (paired Student’s t test, P = 0.2987). Nearly an identical number of subunits was determined using electron microscopy to count catalytic-domain protrusions on wild-type holoenzyme isolated from rat forebrain (predominantly CaMKIIα) [24]. Thus, in solution there is on average 10 V-CaMKIIα subunits in the auto-inhibited holoenzyme.

**Figure 3:**
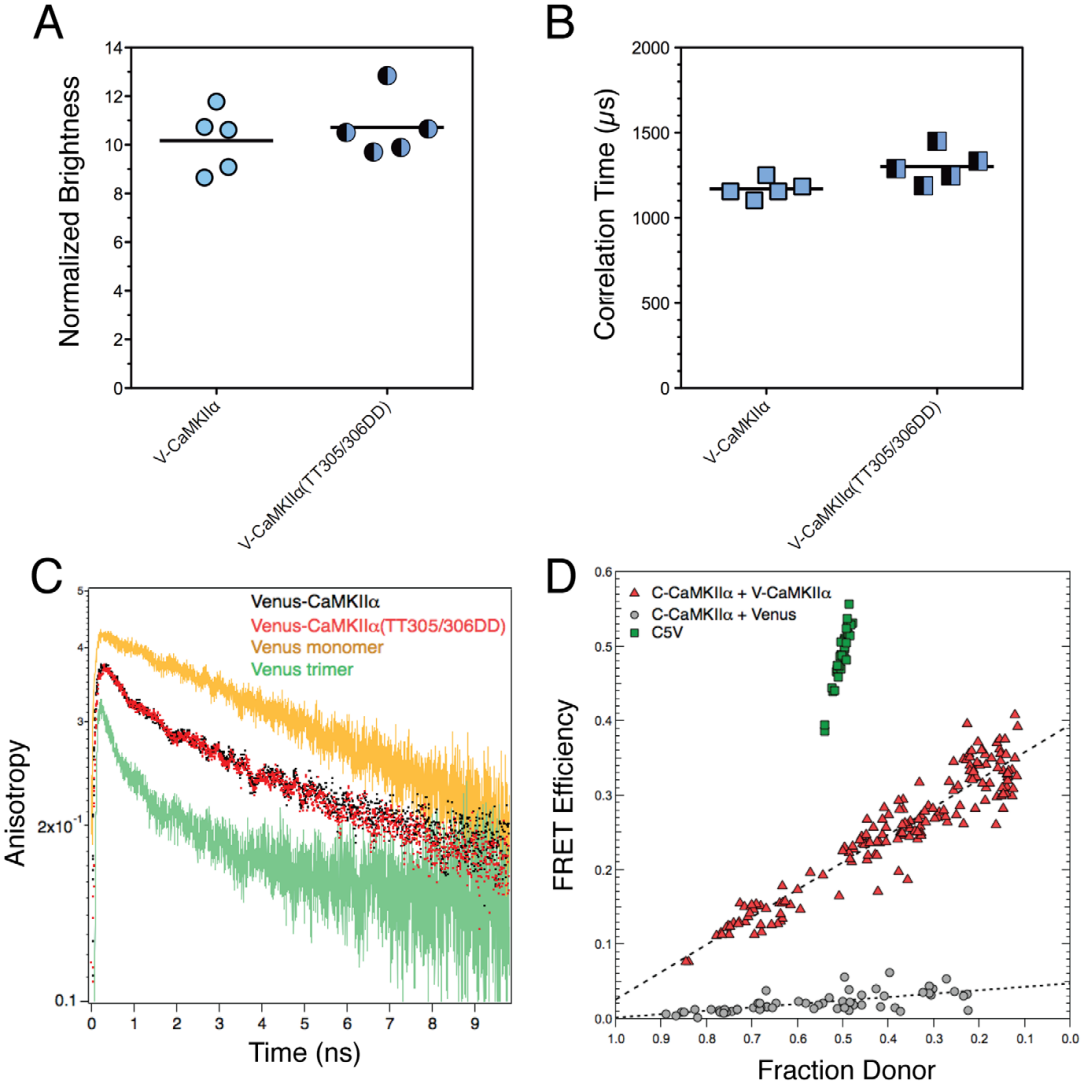
FPFA of CaMKIIα holoenzyme. (A) The normalized brightness for V-CaMKIIα holoenzyme, and mutant that cannot bind Ca^2+^/CaM. Bars represent the means with n = 5. The excitation power used was 10.2 mW. (B) The correlation times for samples in panel A. (C) Average TRA for samples in panel A. TRA traces for Venus monomers (V1) and Venus trimers (V3) from figure 2B are overlaid as a reference, and to illustrate that the Venus-tagged catalytic domains in V-CaMKIIα produce an anisotropy signal most consistent with Venus-dimers. (D) Hetero-FRET analysis of CaMKIIα catalytic domain pairing in living cells. Cells were transfected with DNA constructs encoding CaMKIIα tagged on the catalytic domain with either Cerulean or Venus. Cells transfected with Cerulean-CaMKIIα & free Venus monomers were used as a negative FRET control. Cells transfected with C5V was used as a positive FRET control. Each point is the average FRET efficiency and fraction donor value measured for an individual cell. Dashed lines are linear fits.

The correlation time for V-CaMKIIα holoenzyme was 1.2±0.1 ms (mean±SD, n = 5) and for V-CaMKIIα[TT305/306DD] it was 1.3±0.1 ms (mean±SD, n = 5) (Fig 3B). As expected for a large holoenzyme, these samples diffused much slower than the Venus monomer (Fig 2D). TRA analysis revealed a fast homo-FRET component for V-CaMKIIα and V-CaMKIIα[TT305/306DD] indicative of catalytic domain pairing (Fig 3). Hetero-FRET analysis was used to confirm the existence of catalytic domain pairing in the CaMKIIα holoenzyme, as well as to measure the FRET efficiency detected between fluorescent protein tagged catalytic domains. Cells were transfected with DNA constructs encoding CaMKIIα tagged on the catalytic domain with either Cerulean [44] acting as a FRET donor, or Venus [38] as a FRET acceptor. Cells transfected with Cerulean-CaMKIIα (C-CaMKIIα) & free Venus monomers were used as a negative FRET control, while cells transfected with C5V, a construct with a Cerulean donor tethered to a Venus acceptor using a 5 amino-acid linker [45], [46], was used as a positive FRET control. FRET efficiency values and the fraction donor were measured the following day from live cells using E-FRET microscopy [47], [48]. Cells transfected with C-CaMKIIα & V-CaMKIIα had FRET efficiencies that increased linearly as the donor fraction decreased (Fig 3D). A linear relationship between the observed FRET efficiency when plotted as a function of the donor fraction is indicative of FRET occurring between 1 donor and 1 acceptor [49], and suggests that catalytic domains form pairs within the holoenzyme structure in living cells. The FRET efficiency (the y-intercept when the donor fraction is zero) between fluorescent protein-tagged CaMKIIα catalytic domains was 39.4±0.8%, while the FRET efficiency for our negative control was 5.6±0.8%. The FRET efficiency for the C5V positive control was 48.9±3.8% (mean±SD, n = 34 cells), and C5V’s fraction donor was 0.50±0.01 as expected for a construct with one donor covalently linked to one acceptor.

### FPFA Measurements in Cells

**Figure 4:**
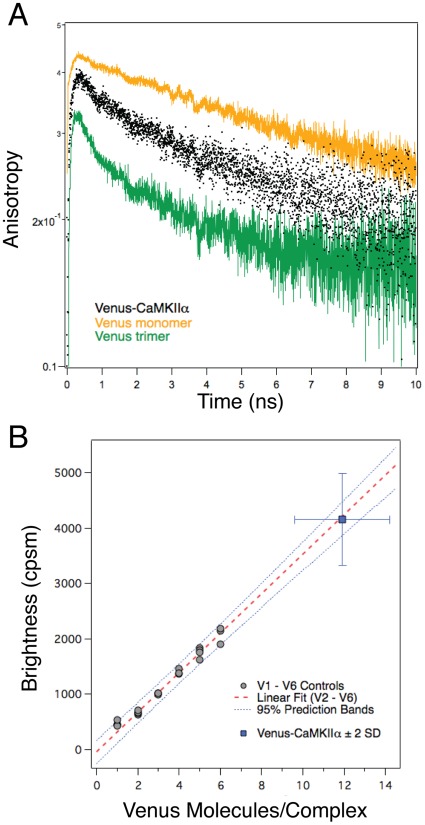
FPFA of V-CaMKIIα expressed in HEK cells. (A) Average TRA for V-CaMKIIα (Black) holoenzyme in cells. TRA traces for Venus monomers (V1, Yellow) and Venus trimers (V3, Green) expressed in HEK cells are also plotted as a reference, and to illustrate that the Venus-tagged catalytic domains in V-CaMKIIα produce an anisotropy signal most consistent with Venus-dimers. The excitation power used was 6 mW. (B) The brightness values for V1–V6 expressed and measured in HEK cells are plotted as a function of the number of Venus molecules in each construct. Each point represents a single cell, and at least 3 cells were measured for each Venus concatemer. Note that the V1 brightness was slightly elevated, presumably due to endogenous autofluorescence. Red dashed line indicates fit to a linear model for V2–V6 with dashed blue lines indicating the 95% confidence bands. The average brightness and normalized brightness of V-CaMKIIα (Blue squares, mean±SD, n = 11 cells) is plotted on the main graph with error bars indicating two standard deviations.

To measure the number of subunits that comprise the CaMKIIα holoenzyme in living cells using FPFA required expressing V-CaMKIIα at low enough concentrations to detect fluctuation, while also allowing sufficient time for protein expression, Venus maturation, and holoenzyme assembly. Because the amount of protein expression at 24 hours could be controlled by titrating the amount of RNA used in a transfection [50], RNA encoding V-CaMKIIα was used for live cell FPFA. Additionally, the laser power for live cell FPFA was reduced to 6 mW to prevent bleaching. The TRA of V-CaMKIIα in cells (Fig 4A) was similar to the signals observed in solution being more depolarized than the V1 monomer, but less depolarized than the V3 Venus trimer (Fig 3C), and together with hetero-FRET experiments in cells (Fig 3D) indicates that catalytic domain pairing occurs in cells as well as in solution. The brightness of cells transfected with RNA encoding the V1–V6 controls are shown in figure 4B. In cells, the brightness of these concatemers was still linear with the number of Venus molecules with the exception that V1 showed a slightly elevated value. This can be attributed to endogenous autofluorescence. The V2–V6 brightness values were well fit to a linear model so a linear interpolation was used to calculate normalized brightness of Venus-CaMKIIα holoenzyme in cells. The normalized brightness for V-CaMKIIα in cells was 11.9±1.2 (mean±SD, n = 11cells) (Fig 4B). Surprisingly, the holoenzyme in cells had approximately 2 more subunits than holoenzyme in solution. The variance of this measurement was twice as large as the 95% prediction bands of the linear fit of our controls, suggesting that in cells V-CaMKIIα has on average 12 subunits, but ranges from 8–14 subunits.

## Discussion

FPFA was validated using two sets of controls. The first set consisted of 6 Venus concatemers. FPFA readily differentiated these molecules by their brightness, and to a limited extent by their anisotropy and correlation time (Fig 2). Importantly, brightness analysis unambiguously determined the number of Venus molecules in each construct (Fig 2E). The second set of controls consisted of 3 Venus dimers separated by different length amino-acid linkers. Here, based on different fluorophore separation distances, anisotropy could differentiate between these molecules (Fig 2F). Thus, FPFA can detect subtle structural differences based on homo-FRET, changes in mass and hydrodynamics using correlation times, while simultaneously monitoring the number of fluorophores in a complex using brightness analysis.

Several potential sources of error in FPFA measurements warrant consideration. First, polarization artifacts can be avoided by measuring the cross-correlation of parallel and perpendicular polarized detectors [39]. Because the molecular brightness of V5V, V17V and V32V yielded comparable values (although their time-resolved anisotropies were visibly different) this conclusion is supported (Fig 2F). A second potential source of error is the impact of *flickering* on brightness measurements [19]. Because fluorescent protein flickering has not been observed with two-photon excitation, flickering should not impact FPFA measurements as implemented here. This conclusion is supported by the observed linear relationship between molecular brightness and the number of Venus molecules in a concatemer (Fig 2E). Finally, for live cell FPFA measurements, auto-fluorescence can corrupt brightness measurements [16], [17]. The apparent brightness of a sample with more than one fluorophore species is thought to be a nonlinear function weighted by the abundance of each fluorescent species multiplied by the square of its brightness [18], [51] (see discussion in Materials and Methods). In typical live cell experiments the fluorophore tag and the wavelength used to excite it are both selected to maximize the fluorescent signal of the fluorophore, and to minimize cellular auto-fluorescence. Under these conditions, the brightness of the sample will typically be much brighter than the brightness of auto-fluorescent species. Even if the concentration of these dim auto-fluorescent species are significantly higher than the concentration of the fluorophore-tagged species, if a sample has or can be tagged with multiple copies of the fluorophore, eventually the contribution of auto-fluorescence to the apparent brightness of the sample will become negligible. This was observed in figure 4B for Venus concatemers.

Using FPFA we show that in cells V-CaMKIIα holoenzyme has on average 12 subunits (Fig 4B), with its catalytic-domains arranged as pairs (Fig 4A). The pairing of catalytic domains in the holoenzyme was previously observed in hippocampal neurons using anisotropy measurements [10], and this structural feature of the auto-inhibited holoenzyme was confirmed using hetero-FRET measurements in cells (Fig 3D). It is unlikely that attached fluorescent proteins caused this dimerization because 1) a monomeric form of Cerulean and Venus were used in all experiments [41], 2) homo-FRET was not observed between Venus monomers (V1, Figs 2B, & 4A), and 3) isolated non-tagged catalytic domains formed dimers in solution [30].

It is likely that these dimers exist specifically in the postulated auto-inhibited extended form of the CaMKIIα holoenzyme [35] because homo-FRET between Venus-tagged catalytic domains was observed in the V-CaMKIIα[TT305/306DD] construct (Fig 3C). As mentioned previously, the TT_305/306_ to DD_305/306_ mutations are expected to disrupt the compact auto-inhibited holoenzyme structure [35] but also block the activation of the enzyme by preventing Ca^2+^/CaM binding [36]. Thus, our FPFA data supports the postulated Ca^2+^/CaM accessible auto-inhibited holoenzyme structure, but with catalytic domains organized as pairs. Presumably, when catalytic domains un-dock from the association domain core they reorganize to form dimers.

Like in living cells, catalytic domain pairing was also observed in freshly prepared homogenates (Fig 3C). In contrast, the number of subunits in the holoenzymes was reduced from 11.9±1.2 in cells to 10.2±1.3 in homogenates. Thus, catalytic domain pairing as observed by homo-FRET occurs in cells as well as *in vitro* and persists despite a reduction in holoenzyme subunits stoichiometry. This would be expected if the reduction in subunits represent a loss of a pair of subunits from the holoenzyme with their paired catalytic domains, rather than a loss of two independent subunits. This type of paired subunit organization has been previously proposed based on subunit stoichiometry distributions from electron microscopy imaging of native holoenzyme [24].

An implicit assumption of using brightness analysis to measure the number of subunits in a CaMKII holoenzyme is that only subunits tagged with a fluorophore are incorporated into a complex. This assumption may not always be warranted, particularly for cells that express a high level of non-tagged endogenous subunits. Under these conditions it is prudent to interpret normalized brightness as a lower limit for the number of subunits in a complex. In these studies for Venus-CaMKIIα expression in HEK cells this is unlikely to be a problem because the CaMKII subunit stoichiometry predicted by FPFA analysis was in good agreement with estimates based on non-tagged native holoenzyme [23], [24]. Nonetheless, a conservative interpretation of our data would conclude that in cells CaMKIIα holoenzyme typically has ≥12 subunits.

The impact of fluorophore-tagging must be considered when interpreting structural findings based on fluorescence. Here FPFA detected 2 distinct V-CaMKIIα protein interactions which were previously observed using untagged-CaMKII. First, in cells V-CaMKIIα subunits assembled to form a dodecamer (Fig 4B), a value similar to *in vitro* estimates using native holoenzyme [23], [24], and *in vivo* measurements based on GFP-tagged CaMKIIα translocation [25]. Second, catalytic domains were arranged as pairs in the holoenzyme (Figs 3C & 4A), consistent with homo-FRET [10], hetero-FRET (Fig 3D), and ultra-centrifugation studies using untagged catalytic domains [30]. Thus, it is unlikely that Venus-tagging caused or significantly perturbed these interactions.

It is worth comparing the relative merits of CaMKIIα holoenzyme structural studies inside living cells based on considering only hetero-FRET data (Fig 3D), only homo-FRET data ([10], and Fig 4A), as compared to considering both homo-FRET and brightness analysis from FPFA (Fig 4A & B). Hetero-FRET analysis (Fig 3D) readily demonstrated that fluorescent protein-tagged CaMKIIα catalytic domains were in close proximity. The ensemble FRET efficiency for the 171 cells transfected with C-CaMKIIα and V-CaMKIIα was 25.2±7.9%, significantly higher than our negative control. Nonetheless, FRET efficiencies ranging from 0 to 40% were observed depending on the relative expression of Cerulean- and Venus-tagged CaMKIIα subunits. Thus, if the fraction donor were not simultaneously measured, as is often the case for many FRET approaches, interpretation of this hetero-FRET data would be problematic. For example, our interpretation of CaMKIIα hetero-FRET in cells, as energy transfer between *paired* donors and acceptors with a FRET efficiency of 39.4±0.8% was based on modeling how the FRET efficiency changed from cell to cell as a function of the fraction donor. While we feel this interpretation is compelling, we also note that these experiments cannot differentiate between holoenzyme structures having a single set of paired subunits, and various other possible configurations comprised of multiple sets of paired subunits, and even our negative control showed a small amount of FRET, perhaps as a result of over expression.

Homo-FRET analysis (Fig 4A) could also readily detect energy transfer between Venus-tagged catalytic domains, again indicating that in cells Venus-tagged catalytic domains are in close proximity to other Venus-tagged catalytic domains. Based on the amplitude of V-CaMKIIα’s fast anisotropy decay component, compared to the anisotropy decays of Venus monomer and trimer controls, it is most likely that V-CaMKIIα’s catalytic domains are organized as pairs [10]. Like the previous hetero-FRET analysis, homo-FRET analysis alone cannot differentiate between a holoenzyme structure having a single set of paired subunits, and configurations comprised of multiple sets of paired subunits. One advantage of homo-FRET over hetero-FRET in that only a single fluorescent protein tagged CaMKII subunit, V-CaMKIIα, was needed for these measurements. Thus, possible errors related to the relative expression of donor and acceptor-tagged subunits are eliminated.

Both homo- and hetero-FRET analysis are susceptible to false positive FRET signals due to over expression of fluorophore-tagged subunits in cells. One advantage of homo-FRET analysis of data collected by FPFA is that the possibility of false positives is almost completely eliminated. For soluble fluorophores, non-specific FRET typically requires concentrations greater than 1 mM [4], [52]. With the FPFA instrumental design reported here this would correspond to having in excess of 210,000 Venus molecules in the 0.35 fl observation volume of our microscope. At these high concentrations fluorescence intensity fluctuations (due to individual fluorophores moving in and out of the observation volume) are too small relative to the average fluorescence intensity to be measured [17]. For this reason, FCS and FPFA measurements, by necessity, are limited to samples with concentrations much less than 1 µM (∼210 molecules). Furthermore, to avoid TCSPC pile-up errors [13] in time-resolved anisotropy measurements, samples for FPFA was further restricted to those with count-rate less than 100,000 cps. Within these limits, the maximum number of Venus molecules in the observation volume that we could measure is ∼120 (∼570 nM), but was typically much smaller. For example, in figure 1D the number of Venus molecules in the observation volume was ∼65. Thus, observing non-specific FRET in a FPFA experiment is highly unlikely. Another major advantage of FPFA analysis over both hetero- and homo-FRET analysis is that FPFA simultaneously measures normalized brightness. Thus we can deduce that the dimeric homo-FRET signal we observed in cells for V-CaMKIIα (Fig 4A) was coming from protein complexes having on average at least 12 subunits (Fig 4B). This observation eliminates the possibility that tagging CaMKIIα on its N-terminus (catalytic domain) prevented holoenzyme oligomerization via its association domain. We conclude that the advantages of being able to simultaneously measuring homo-FRET, brightness, and correlation time to study protein interactions in living cells justifies the use of FPFA over other FRET approaches, and in addition to being a useful tool for understanding the structure of the CaMKII holoenzyme; FPFA can also be used to study protein interactions in other complexes as well.

## Materials and Methods

### Cell Culture, Transfection and Homogenate Preparation

HEK 293 cells (ATCC) were cultured as a monolayer in a T-75 Flask (Corning) in a humidified incubator containing 5% CO_2_ in air at 37°C in High Glucose DMEM media containing L-Glutamine, sodium pyruvate and 10% fetal bovine serum (all from Gibco). A day prior to FPFA measurement, cells were resuspended using TrypLE Express (Invitrogen) and washed with DPBS (with calcium and magnesium, Mediatech). For in vitro measurements, plasmid DNA encoding Venus-tagged constructs (typically 1 µg/250,000 cells) were transfected into the cells using electroporation (Digital Bio/BTX MicroPorator). Transfected cells were plated on 60 mm culture dishes (Falcon) and incubated overnight. On the following day, cells were harvested and lysed using passive lysis buffer (Promega). Homogenates were centrifuged at 100,000×g for 1 hour to remove membranes and particulate matter. Supernatants were diluted for FPFA to yield a photon count rate between ∼20,000 cps (>25× the dark count rate) and <100,000 cps (to avoid TCSPC pile-up artifacts [13]). The clarified homogenates were then loaded into 35 mm glass bottom dishes (MatTek) and micro- and macro-times were measured by FPFA the same day. For live-cell measurements, cells were transfected with RNA encoding Venus-tagged constructs since the amount of protein expression after 24 hours could be controlled by titrating the amount of RNA used during transfection, typically around 500 µg of RNA/250,000 cells was used. Transfected cells were plated on 35 mm glass bottom dishes and incubated overnight in phenol-red free DMEM media and FPFA was measured the next day.

### Molecular Biology and Immunoblot Analysis

DNA clones encoding V1 (Addgene ID 277794), V2/V5V (Addgene ID 29423), V3 (Addgene ID 27814), V4 (Addgene ID 29425), V5 (Addgene ID 29426), V6 (Addgene ID 27813), V17V (Addgene ID 29424), V32V (Addgene ID 29561), and V-CaMKIIα (Addgene ID 29428) are available from Addgene (http://www.addgene.org/Steven_Vogel#). DNA clones encoding V-CaMKIIα [TT305/306DD], and C-CaMKIIα, were generated as follows: Sense (5′-GGAGCCATCCTCGACGACATGCTGGCCACCAGG-3′) and antisense (5′-CCTGGTGGCCAGCATGTCGTCGAGGATGGCTCC-3′) primers were used to perform site directed mutagenesis PCR (as previously described [10]) to generate V-CaMKIIα[TT305/306DD]. Note that the mutated bases are underlined in the primer sequences. C-CaMKIIα was generated by excising the open reading frame of mouse CaMKIIα from V-CaMKIIα (Addgene ID 29428) and inserting it into a Sal1/BamH1 digested Cerulean C1 (Addgene ID 27796). The resulting C-CaMKIIα construct was confirmed by sequencing. For RNA transfection, DNA encoding full length V-CaMKIIα was excised and inserted into the Prn2 vector [50]. This clone was digested using Not-1 and the linearized plasmid was used to generate cRNA using a mMachine mMessage RNA kit (Ambion).

#### Immunoblot analysis

HEK 293 cells were transfected with plasmid DNA encoding the V1–V6 constructs. Cells (80–100% confluent) expressing Venus concatamers were scrapped off the dish, washed twice with PBS, and pelleted. The pellet was then lysed in denaturing Laemmli buffer (BioRad) with 1% SDS and β-Mercaptoethanol and heated to 90 C° for 10 minutes. Cell debris was removed by centrifugation (20,000×g for 10 mins), and 20 µl of the solution was loaded into a precast gradient SDS PAGE gel (4–20%; BioRad). Immunoblotting with a GFP specific antibody (NeuroMab Anti-GFP N86/8) was performed as previously described [42].

### Fluorescence Polarization and Fluctuation Analysis Experimental Setup

An 80-MHz, 200-fs mode locked Ti:sapphire laser (Coherent Chameleon Ultra-2) was tuned to 950 nm to provide pulsed two-photon excitation. The power of the laser beam was adjusted using a variable attenuator consisted of a half-wave plate and a Glan-laser polarizer (Thorlabs). The excitation beam was filtered and expanded using a spatial filter system (KT310, Thorlabs), and then passed through a near-IR linear polarizer (100,000∶1 extinction ratio, Thorlabs) to enter the back port of a Zeiss Axio Observer microscope. A multiphoton short-pass dichroic beamsplitter (FF670-SDi01-25×36, Semrock) was used to reflect the excitation beam to a Zeiss 63×−1.2 NA water objective (back aperture slightly overfilled) that focused the beam to a diffraction-limited spot (∼ 0.4 µm in diameter). Fluorescence from the observation volume in the sample was filtered through a BG39 filter (to block residual near-IR photons), a high throughput band-pass filter (FF01-540/50–25, Semrock), and then guided to a polarizing beam splitter (Thorlabs) that was augmented with two orthogonally oriented linear polarizers (Thorlabs) to increase the extinction ratio. At the beam splitter, parallel and perpendicular emitted photons were separated and focused onto two HPM-100-40 hybrid detectors (Becker & Hickl). The dark count rate for these detectors was typically 400–750 cps at room temperature. Photons detected by each detector were processed by a SPC-132 TCSPC card (Becker & Hickl). The SPC-132 recorded both micro- and macro-time for each parallel and perpendicular detected photon. For synchronization between excitation pulses and detected photons, a small fraction of the excitation beam was extracted and focused onto a fast photodiode that was powered by battery to avoid crosstalk. Note that all optics used in the excitation pathway was selected to minimize group delay dispersion.

SPCM software (Becker & Hickl) running in FIFO mode was used for data acquisition and to calculate time-resolved fluorescence and auto-/cross-correlation functions from measured micro- and macro-time data, respectively. Excitation power was kept low (typically 10.2 mW in vitro and 6 mW in living cells) to avoid bleaching during acquisition (∼120 s in vitro and 20 s in living cells). For each homogenate, three to five replicate measurements were performed and these were averaged for each point. For live-cell measurements ten to fifteen replicate measurements were averaged for each cell. All measurements were performed at room temperature.

### Time Resolved Anisotropy and Lifetime Analysis

Time-resolved anisotropy was calculated based on fluorescence decay of parallel and perpendicular channels using the following equation [10], [11]:
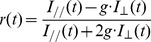
(1)where I_//_(t) and I_⊥_(t) are fluorescence intensity of parallel and perpendicular channels (dark noise subtracted) respectively, and g is the instrument correction factor which for our microscope had a value of 1 as determined by calibration using fluorescein tail fitting.

The total time-dependent fluorescence intensity decay or *lifetime* was calculated using the following relationship [13].
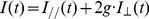
(2)


### Polarized Fluorescence Fluctuation Analysis

For FPFA, a cross-correlation curve is fitted to a single component three-dimensional Gaussian function [17] G(τ) to estimate the values <N>, the average number of fluorescent molecules in the excitation volume, and τ_D_, the correlation time, the average time that a molecule spends in the detection volume:
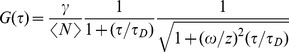
(3)Where ω and z, are the radial and axial beam waists respectively, and the constant γ has a value of 0.35 for a two-photon three-dimensional Gaussian PSF [17].

The molecular brightness η is the average number of photon emitted per second per molecule (cpsm):
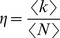
(4)where *<k>* is the average photon count rate recorded during data acquisition.

The normalized brightness, ρ, of a Venus-tagged protein complex was determined by dividing the molecular brightness (η_complex_) of a complex composed of Venus-tagged subunits, by the molecular brightness of a Venus monomer (η_Venus_):
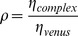
(5)Note that η_complex_ and η_Venus_ should be measured using similar conditions (primarily using the same laser excitation power, filters, and optics), and that η_Venus_ can be measured by running a Venus monomer control. In cells, where auto-fluorescence was an issue, the normalized brightness was calculated using the measured slope (*m*) and y-intercept (*b*) of the linear portion of the V1–V6 brightness curve:
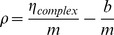
(6)With two-photon excitation the relationship between correlation time τD and the diffusion coefficient D is given by [53]:
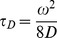
(7)


### Calibration

The instrument correction factor *g* for calculating time-resolved anisotropy (equation 1) was measured using tail-fitting [11] of fluorescein samples and found to be 1. At high pH, fluorescein has a constant quantum yield and its diffusion coefficient D is 300 µm^2^/s at room temperature [17]. Thus, equation 7 can be used to estimate the value of ω (at a specific excitation power) by measuring fluorescein’s correlation time (at the same power). For example, with D = 300 µm^2^/s, and a measured correlation times of 74.8±9.3 µs (n = 3), the value of ω was 424±26 nm with 10.2 mW excitation power (at 950 nm). The ratio ω/z (equations 3 and 4) was measured by global fitting (to equation 3) of cross-correlation curves obtained from known dilutions of fluorescein. In this calibration it is assumed that with constant excitation power for all fluorescein dilutions, only the value <N> will change with dilution. At 10.2 mW excitation power the ω/z ratio was 0.15±0.00, and taken together with our estimate for ω predicts a z value of 2.8±0.2 µm. The validity of this calibration procedure was confirmed by measuring the diffusion coefficient of Venus monomers under identical conditions. Using ω = 424 nm, and ω/z = 0.15 the measured correlation time for Venus monomers with 10.2 mW excitation power was 345±16 µs (n = 3). This, corresponds to a diffusion coefficient for Venus monomers in solution of 65±3 µm^2^/s (n = 3) in good agreement with the diffusion coefficient measured for GFP [51]. Because fluorescein was poorly excited with 6 mW excitation power at 950 nm, calibration for live cell FPFA used a slightly different procedure. Global fitting of cross-correlation curves obtained from dilutions of Venus monomers were used to measure the ratio ω/z (0.10±0.00), as well as the correlation time of Venus monomers (299±65 µs, n = 3). These values indicated that with 6 mW excitation power ω = 367±85 nm and z was 3.7±0.9 µm. Note that these values had large errors because of the low excitation power. We used ω and z values measured at 6 and 10.2 mW excitation power to determine if the two-photon observation volume changed appreciably with increased excitation power. The two-photon observation volume (V) at any specific excitation power can be calculated using the following equation [17]:
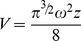
(8)Accordingly, the observation volume with 10.2 mW excitation power was 0.35 fl, only slightly larger than the volume measured at lower power (6 mW, 0.34 fl). Note that these volumes, and the value of <N> from a FPFA measurement can be used to calculate the concentration of Venus or of Venus-tagged protein complexes, a key factor for determining if non-specific FRET (due to molecular crowding) can occur.

### Hetero-FRET Measurements

DNA encoding CaMKIIα tagged on the catalytic domain with Cerulean [44] acting as a FRET donor, and Venus [38] as a FRET acceptor were transfected into HeLa cells (ATCC). Cells transfected with Cerulean-CaMKIIα & free Venus monomers were used as a negative FRET control, and cells transfected with C5V [45] was used as a positive control. Images were acquired on an automated wide-field microscope and FRET analysis was performed using the E-FRET method [48] calibrated with Cerulean and Venus FRET standards [45]. Each point is the average FRET efficiency and fraction donor value measured for an individual cell. Dashed lines are linear fits. A linear relationship between the observed FRET efficiency (E_obs_) when plotted as a function of the fraction donor (*p*) indicates that FRET is only occurring between 1 donor and 1 acceptor [49]. This linear relationship can be understood as follows: If CaMKII subunits form random pairs based only on their abundance, then if *p* is the fraction of Cerulean-tagged CaMKII subunits in the population, then (1-*p*) will be the fraction of Venus-tagged CaMKII subunits in the population. The random pairing of Cerulean- and Venus-tagged catalytic domains can be modeled using a binomial distribution. In a population the fraction of Cerulean-tagged CaMKII subunits paired with other Cerulean-tagged CaMKII subunits will be *p^2^*, and each of the 2 Cerulean molecules in these pairings will have a FRET efficiency of zero because they are both not pared with a Venus acceptor. Similarly, the fraction of Venus-tagged CaMKII subunits paired with another Venus-tagged CaMKII subunits will be *(1-p)^2^*, but because these pairs lack donors, they have no direct impact on the FRET efficiency of the population. Finally, the fraction of Cerulean-tagged CaMKII subunits paired with Venus-tagged CaMKII subunits will be *2p(1-p)*, and because each has a single donor (and acceptor), they will each have a FRET efficiency of E. Thus the observed ensemble FRET efficiency for the population E_obs_ as a function of the fraction donor (*p*) will be:
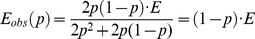
(9)Note, that E_obs_ is a linear function of *p*, and that the slope and *y*-intercept (when *p* = 0) are both equal to the FRET efficiency between a single donor and acceptor.

### The Impact of Autofluorescence on Brightness Measurements for Live Cell FPFA

For live cell FCS measurements, auto-fluorescence and other background light sources can impact on brightness measurements [16], [17], [18], [51]. In addition to the fluorophore of interest, endogenous fluorophores can also emit photons resulting in an altered *apparent* brightness. This problem can also occur for FPFA based brightness measurements. Corrections for two different types of background signals have been described, these are: 1. Background signals that only alter the photon-count rate, and 2. Background fluorescence from endogenous fluorophores that fluctuate. For background signals that only alter the photon-count rate the apparent molecular brightness (*η_app_*) of the sample is [16], [18]:
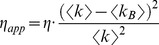
(10)Where *η* is the brightness of the exogenous fluorophore, *<k>* is the average measured count rate (sample plus background), and *<k_B_>* is the average measured count rate of the background. The apparent brightness of a sample with fluctuating endogenous fluorophores is [18], [51]:
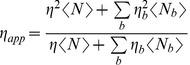
(11)Where η and <N> are the molecular brightness and the average number of exogenous fluorophore molecules in the observation volume, and ηb and <Nb> are the molecular brightness and the average number of molecules in the observation volume of the bth fluorescence background species. In living cells it is possible that both of these types of background signals can adversely contribute to the apparent brightness. Furthermore, the relative impact of these types of background signals on the apparent brightness is rarely known. Moreover, many of the factors in equation 11 are either difficult to measure or are themselves unknown, and it is worth noting that the validity of these corrections has not been rigorously tested. An alternative empirical strategy for interpreting the apparent brightness of a complex composed of FP-tagged subunits, in terms of subunit stoichiometry, is to compare an unknown samples brightness to the measured brightness of a series of FP-concatemers expressed in the same cell type, such as the use of the V1– V6 series used in this study. The underlying assumption for this calibration strategy is that the brightness, abundance and fluctuation behavior of the sources of cellular background signals are not altered by the expression of different FP-tagged constructs. Under ideal conditions, experimental samples will have brightness values falling within the range of a control set (1–6 subunits in this instance). Under these conditions control brightness values can be used directly as a standard curve to interpret the brightness of an unknown. For samples with an apparent brightness greater than the largest concatemer in a control set (in this example those with >6 subunits) the subunit stoichiometry of the sample can be estimated by extrapolating brightness values based on the standard brightness curve. While extrapolations beyond control values is not ideal, by using proper error propagation [54] errors in interpretation can be minimized.

### Curve Fitting and Statistics

IGOR Pro software (Vs 6.22) was used for standard and global fitting of time-resolved anisotropy, cross-correlation curves, and linear fits for brightness controls. GraphPad Prism 5 was used to calculate means and standard deviations (SD). Values are presented throughout the text as mean±SD, deviations of ±0.00 indicate a value of less than 0.005, while deviations of ±0.0 indicate a value of less than 0.05. GraphPad Prism was also used to calculate Student’s t-test, which was paired and two-tailed.

## References

[1] Förster Th (1948). Intermolecular energy migration and fluorescence.. Annalen der Physik.

[2] Sekar RB, Periasamy A (2003). Fluorescence resonance energy transfer (FRET) microscopy imaging of live cell protein localizations.. J Cell Biol.

[3] Jares-Erijman EA, Jovin TM (2006). Imaging molecular interactions in living cells by FRET microscopy.. Curr Opin Chem Biol.

[4] Vogel SS, Thaler C, Koushik SV (2006). Fanciful FRET..

[5] Clegg RM, Gadella TWJ (2009). Forster resonance energy transfer -FRET what it is, why do it, and how it’s done.. FRET and FLIM Techniques.

[6] Piston DW, Kremers GJ (2007). Fluorescent protein FRET: the good, the bad and the ugly.. Trends in Biochemical Sciences.

[7] Stryer L, Haugland RP (1967). Energy transfer: a spectroscopic ruler.. Proc Natl Acad Sci U S A.

[8] Gautier I, Tramier M, Durieux C, Coppey J, Pansu RB (2001). Homo-FRET microscopy in living cells to measure monomer-dimer transition of GFP-tagged proteins.. Biophys J.

[9] Sharma P, Varma R, Sarasij RC, Ira, Gousset K (2004). Nanoscale organization of multiple GPI-anchored proteins in living cell membranes.. Cell.

[10] Thaler C, Koushik SV, Puhl HL, 3rd, Blank PS, Vogel SS (2009). Structural rearrangement of CaMKIIα catalytic domains encodes activation.. Proc Natl Acad Sci U S A.

[11] Vogel SS, Thaler C, Blank PS, Koushik SV, Periasamy A, Clegg RM (2010). Time Resolved Fluorescence Anisotropy.. FLIM Microscopy in Biology and Medicine.

[12] Bader AN, Hofman EG, Voortman J, en Henegouwen PM, Gerritsen HC (2009). Homo-FRET imaging enables quantification of protein cluster sizes with subcellular resolution.. Biophys J.

[13] Becker W (2005). Advanced Time-Correlated Single Photon Counting Techniques; Castleman AW, Toennies JP, Zinth W, editors..

[14] Magde D, Elson EL, Webb WW (1974). Fluorescence correlation spectroscopy. II. An experimental realization.. Biopolymers.

[15] Elson EL, Webb WW (1975). Concentration correlation spectroscopy: a new biophysical probe based on occupation number fluctuations.. Annu Rev Biophys Bioeng.

[16] Schwille P, Haupts U, Maiti S, Webb WW (1999). Molecular dynamics in living cells observed by fluorescence correlation spectroscopy with one- and two-photon excitation.. Biophys J.

[17] Muller JD, Chen Y, Gratton E (2003). Fluorescence correlation spectroscopy.. Methods Enzymol.

[18] Thompson NL, Lakowicz JR (1991). Fluorescence Correlation Spectroscopy.. Topics in Fluorescence Spectroscopy.

[19] Chen Y, Johnson J, Macdonald P, Wu B, Mueller JD (2010). Observing protein interactions and their stoichiometry in living cells by brightness analysis of fluorescence fluctuation experiments.. Methods Enzymol.

[20] Sahoo H, Schwille P (2011). FRET and FCS–friends or foes?. Chemphyschem.

[21] Weidtkamp-Peters S, Felekyan S, Bleckmann A, Simon R, Becker W (2009). Multiparameter fluorescence image spectroscopy to study molecular interactions.. Photochem Photobiol Sci.

[22] Lisman J, Schulman H, Cline H (2002). The molecular basis of CaMKII function in synaptic and behavioural memory.. Nat Rev Neurosci.

[23] Hudmon A, Schulman H (2002). Structure-function of the multifunctional Ca^2+^/calmodulin-dependent protein kinase II.. Biochem J.

[24] Kanaseki T, Ikeuchi Y, Sugiura H, Yamauchi T (1991). Structural features of Ca^2+^/calmodulin-dependent protein kinase II revealed by electron microscopy.. J Cell Biol.

[25] Shen K, Teruel MN, Subramanian K, Meyer T (1998). CaMKIIβ functions as an F-actin targeting module that localizes CaMKIIα/β heterooligomers to dendritic spines.. Neuron.

[26] Kim SA, Heinze KG, Bacia K, Waxham MN, Schwille P (2005). Two-photon cross-correlation analysis of intracellular reactions with variable stoichiometry.. Biophys J.

[27] Sanabria H, Digman MA, Gratton E, Waxham MN (2008). Spatial diffusivity and availability of intracellular calmodulin.. Biophys J.

[28] Miller SG, Kennedy MB (1985). Distinct forebrain and cerebellar isozymes of type II Ca^2+^/calmodulin-dependent protein kinase associate differently with the postsynaptic density fraction.. J Biol Chem.

[29] Hoelz A, Nairn AC, Kuriyan J (2003). Crystal structure of a tetradecameric assembly of the association domain of Ca^2+^/calmodulin-dependent kinase II.. Mol Cell.

[30] Rellos P, Pike AC, Niesen FH, Salah E, Lee WH (2010). Structure of the CaMKIIδ/calmodulin complex reveals the molecular mechanism of CaMKII kinase activation.. PLoS Biol.

[31] De Koninck P, Schulman H (1998). Sensitivity of CaM kinase II to the frequency of Ca^2+^ oscillations.. Science.

[32] Hanson PI, Meyer T, Stryer L, Schulman H (1994). Dual role of calmodulin in autophosphorylation of multifunctional CaM kinase may underlie decoding of calcium signals.. Neuron.

[33] Kolb SJ, Hudmon A, Ginsberg TR, Waxham MN (1998). Identification of domains essential for the assembly of calcium/calmodulin-dependent protein kinase II holoenzymes.. J Biol Chem.

[34] Rosenberg OS, Deindl S, Sung RJ, Nairn AC, Kuriyan J (2005). Structure of the autoinhibited kinase domain of CaMKII and SAXS analysis of the holoenzyme.. Cell.

[35] Chao LH, Stratton MM, Lee IH, Rosenberg OS, Levitz J (2011). A mechanism for tunable autoinhibition in the structure of a human Ca^2+^/calmodulin- dependent kinase II holoenzyme.. Cell.

[36] Takao K, Okamoto K, Nakagawa T, Neve RL, Nagai T (2005). Visualization of synaptic Ca^2+^/calmodulin-dependent protein kinase II activity in living neurons.. J Neurosci.

[37] Wang P, Wu YL, Zhou TH, Sun Y, Pei G (2000). Identification of alternative splicing variants of the beta subunit of human Ca(2+)/calmodulin-dependent protein kinase II with different activities.. FEBS Lett.

[38] Nagai T, Ibata K, Park ES, Kubota M, Mikoshiba K (2002). A variant of yellow fluorescent protein with fast and efficient maturation for cell-biological applications.. Nat Biotechnol.

[39] Enderlein J, Gregor I, Patra D, Fitter J (2004). Art and artefacts of fluorescence correlation spectroscopy.. Curr Pharm Biotechnol.

[40] Kremers GJ, Goedhart J, Gadela TWJ (2009). Visible fluorescent proteins for FRET.. FRET and FLIM Techniques.

[41] Zacharias DA, Violin JD, Newton AC, Tsien RY (2002). Partitioning of lipid-modified monomeric GFPs into membrane microdomains of live cells.. Science.

[42] Sarkar P, Koushik SV, Vogel SS, Gryczynski I, Gryczynski Z (2009). Photophysical properties of Cerulean and Venus fluorescent proteins.. J Biomed Opt.

[43] Shen K, Meyer T (1999). Dynamic control of CaMKII translocation and localization in hippocampal neurons by NMDA receptor stimulation.. Science.

[44] Rizzo MA, Springer GH, Granada B, Piston DW (2004). An improved cyan fluorescent protein variant useful for FRET.. Nat Biotechnol.

[45] Koushik SV, Chen H, Thaler C, Puhl HL, 3rd, Vogel SS (2006). Cerulean, Venus, and VenusY67C FRET reference standards.. Biophys J.

[46] Thaler C, Koushik SV, Blank PS, Vogel SS (2005). Quantitative multiphoton spectral imaging and its use for measuring resonance energy transfer.. Biophys J.

[47] Zal T, Gascoigne NR (2004). Photobleaching-corrected FRET efficiency imaging of live cells.. Biophys J.

[48] Chen H, Puhl HL, 3rd, Koushik SV, Vogel SS, Ikeda SR (2006). Measurement of FRET efficiency and ratio of donor to acceptor concentration in living cells.. Biophys J.

[49] Veatch W, Stryer L (1977). The dimeric nature of the gramicidin A transmembrane channel: conductance and fluorescence energy transfer studies of hybrid channels.. J Mol Biol.

[50] Williams DJ, Puhl HL, Ikeda SR (2011). A Simple, Highly Efficient Method for Heterologous Expression in Mammalian Primary Neurons Using Cationic Lipid-mediated mRNA Transfection.. Front Neurosci.

[51] Chen Y, Muller JD, Ruan Q, Gratton E (2002). Molecular brightness characterization of EGFP in vivo by fluorescence fluctuation spectroscopy.. Biophys J.

[52] Fung BK, Stryer L (1978). Surface density determination in membranes by fluorescence energy transfer.. Biochemistry.

[53] Berland KM, So PT, Gratton E (1995). Two-photon fluorescence correlation spectroscopy: method and application to the intracellular environment.. Biophys J.

[54] Bevington PR, Robinson DK (1992). Data reduction and error analysis for the physical sciences..

